# Expression of a recombinant FLT3 ligand and its emtansine conjugate as a therapeutic candidate against acute myeloid leukemia cells with FLT3 expression

**DOI:** 10.1186/s12934-021-01559-6

**Published:** 2021-03-10

**Authors:** Dengyang Zhang, Yao Guo, Yuming Zhao, Liuting Yu, Zhiguang Chang, Hanzhong Pei, Junbin Huang, Chun Chen, Hongman Xue, Xiaojun Xu, Yihang Pan, Ningning Li, Chengming Zhu, Zhizhuang Joe Zhao, Jian Yu, Yun Chen

**Affiliations:** 1 Edmond H. Fischer Translational Medical Research Laboratory, Scientific Research Center, The Seventh Affiliated Hospital, Sun Yat-Sen University, Shenzhen, 518107 Guangdong China; 2 Department of Pediatrics, The Seventh Affiliated Hospital, Sun Yat-Sen University, Shenzhen, 518107 Guangdong China; 3 Department of Hematology, The Seventh Affiliated Hospital, Sun Yat-Sen University, Shenzhen, 518107 Guangdong China; 4 Department of Pathology, University of Oklahoma Health Sciences Center, 1100 N. Lindsay, Oklahoma City, OK 73104 USA; 5 Beijing Advanced Innovation Center for Biomedical Engineering, Beihang University, Beijing, 100083 China

**Keywords:** FLT3, FL, AML, DM1, Targeted therapy

## Abstract

**Background:**

Most patients with acute myeloid leukemia (AML) remain uncurable and require novel therapeutic methods. Gain-of-function FMS-like tyrosine kinase 3 (FLT3) mutations are present in 30–40% of AML patients and serve as an attractive therapeutic target. In addition, FLT3 is aberrantly expressed on blasts in > 90% of patients with AML, making the FLT3 ligand-based drug conjugate a promising therapeutic strategy for the treatment of patients with AML. Here, *E. coli* was used as a host to express recombinant human FLT3 ligand (rhFL), which was used as a specific vehicle to deliver cytotoxic drugs to FLT3 + AML cells.

**Methods:**

Recombinant hFL was expressed and purified from induced recombinant BL21 (DE3) *E. coli*. Purified rhFL and emtansine (DM1) were conjugated by an *N*-succinimidyl 3-(2-pyridyldithio)propionate (SPDP) linker. We evaluated the potency of the conjugation product FL-DM1 against FLT3-expressing AML cells by examining viability, apoptosis and the cell cycle. The activation of proteins related to the activation of FLT3 signaling and apoptosis pathways was detected by immunoblotting. The selectivity of FL-DM1 was assessed in our unique HCD-57 cell line, which was transformed with the FLT3 internal tandem duplication mutant (FLT3-ITD).

**Results:**

Soluble rhFL was successfully expressed in the periplasm of recombinant *E. coli*. The purified rhFL was bioactive in stimulating FLT3 signaling in AML cells, and the drug conjugate FL-DM1 showed activity in cell signaling and internalization. FL-DM1 was effective in inhibiting the survival of FLT3-expressing THP-1 and MV-4-11 AML cells, with half maximal inhibitory concentration (IC_50_) of 12.9 nM and 1.1 nM. Additionally, FL-DM1 induced caspase-3-dependent apoptosis and arrested the cell cycle at the G2/M phase. Moreover, FL-DM1 selectively targeted HCD-57 cells transformed by FLT3-ITD but not parental HCD-57 cells without FLT3 expression. FL-DM1 can also induce obvious apoptosis in primary FLT3-positive AML cells ex vivo*.*

**Conclusions:**

Our data demonstrated that soluble rhFL can be produced in a bioactive form in the periplasm of recombinant *E. coli*. FL can be used as a specific vehicle to deliver DM1 into FLT3-expressing AML cells. FL-DM1 exhibited cytotoxicity in FLT3-expressing AML cell lines and primary AML cells. FL-DM1 may have potential clinical applications in treating patients with FLT3-positive AML.

**Supplementary Information:**

The online version contains supplementary material available at 10.1186/s12934-021-01559-6.

## Background

Acute myeloid leukemia (AML) is a heterogeneous hematological malignancy characterized by disruption of differentiation and uncontrolled proliferation of myeloid blasts [1]. The therapeutic outcome has been improved in some patients with cytotoxic chemotherapies, but there is still no effective treatment for patients with adverse prognoses [2, 3]. Novel therapeutic methods are urgently needed for the treatment of AML. Due to an improved understanding of the molecular pathogenesis of AML, several molecular targets have been identified in recent years [4]. Among them, FMS-like tyrosine kinase 3 (FLT3) is one of the most important genes involved in the initiation and progression of AML [5].

FLT3 is a single transmembrane type-III receptor tyrosine kinase that consists of an extracellular Ig-like domain, a transmembrane domain, a juxtamembrane domain and a tyrosine kinase domain. It is exclusively expressed in hemopoietic progenitors. Upon binding of the FL dimer, FLT3 dimerizes and transphosphorylates several tyrosine residues on the tyrosine kinase domain. The phosphorylated tyrosine domains can recruit Src homology domain− (SH2−) or phosphotyrosine-binding domain− (PTB−) containing adaptors and phosphorylate downstream signaling proteins, such as AKT, MAPK, STAT5, and SFK family members. Such signaling can result in anti-apoptosis and cell survival and proliferation [6]. FLT3 is a promising therapeutic target in AML. It is expressed in more than 90% of blasts from AML patients [7–9]. The total FLT3 expression level in the bone marrow of AML patients is 5–6 times higher than that in healthy donors [7]. These properties can reduce the undesirable side effects of FLT3-targeted drugs while maximizing the cytotoxic effect [10, 11].

Extensive studies have focused on developing drugs targeting FLT3. Tyrosine kinase inhibitors targeting FLT3 have been approved by the FDA for the treatment of newly diagnosed adult AML patients [12] and are also being evaluated in clinical trials [13]. Monoclonal antibodies targeting FLT3 are also being evaluated in preclinical studies or clinical trials [14–16]. Protein drug conjugates, which can eliminate cancer cells directly via the conjugated drug [17, 18], may serve as improved targeted therapy methods for AML. Soluble FL (FL ectodomain) is the endogenous ligand of FLT3 and represent an ideal “trojan horse” to deliver cytotoxic drugs into FLT3-expressing cells. Emtansine (DM1), a modified form of maytansine that binds to tubulin and disrupts mitosis by inhibiting microtubule assembly [19], has the same inhibitory mechanism as vincristine, which is an approved medication for the treatment of AML. DM1 is well known as a highly potent cytotoxic drug [20] and is mainly used as a conjugate payload in antibody drug conjugate (ADC) therapeutics [19, 21, 22]. The first FDA-approved DM1-containing drug, ado-trastuzumab emtansine, significantly prolongs progression-free survival in relapsed patients with human epidermal growth factor receptor 2 (HER2)-positive metastatic breast cancer [21]. Therefore, the combination of FL and DM1 may serve as a possible targeted drug for FLT3-positive AML.

Soluble FL has three disulfide bond knots, which make it difficult to directly express FL as a soluble and bioactive protein in the cytoplasm of *E. coli*. Recombinant hFL has been expressed in yeast as a secretory protein [23] and in *E. coli* as an inclusion body [24]. Here, we expressed soluble rhFL in the periplasm of *E. coli*. Purified rhFL was used to develop a FL-based drug conjugate. DM1 was conjugated to rhFL, and the product, FL-DM1, was evaluated to determine its cytotoxicity and selectivity in cell-based assays. The drug showed potent toxicity and selectivity towards the FLT3-expressing AML cell line and primary AML cell. This FL-drug conjugate may have important clinical applications in treating AML patients with FLT3-expression.

## Materials and methods

### Cell lines and reagents

MV-4-11 (ATCC, CRL-9591) and THP-1 (ATCC, TIB-202) cells were stored in our laboratory. HCD-57 cells and HCD-57 cells transformed with FLT3-ITD (HCD-57/FLT3-ITD) were previously produced and stored in our laboratory [25]. Blood samples of AML patients were collected in the Seventh Affiliated Hospital, Sun Yat-sen University. Primary AML cells were isolated from bone marrows of AML patients by using density-gradient centrifugation with Ficoll-Paque PLUS (GE Healthcare Life Sciences, PA, USA). Patients were provided written informed consent using a protocol approved by the Institutional Review Board of The Seventh Affiliated Hospital, Sun Yat-sen University, in accordance with the Declaration of Helsinki. BL21 (DE3) *E. coli* and pET20 were obtained from Merck (USA). DM1, SPDP and sulfo-SMCC (sulfosuccinimidyl-4-(*N*-maleimidomethyl)cyclohexane-1-carboxylate) were purchased from MCE (USA). Anti-FL polyclonal antibody and rhFL of *E. coli* origin were purchased from PeproTech (USA).

### Protein expression and purification

The human FL ectodomain gene (corresponding to amino acids 27–184) was cloned by specific primers (shown below) through PCR from THP-1 cDNA. The gene sequence of the FL ectodomain protein with a C-terminal 6 × His tag was verified by Sanger sequencing and cloned in frame into the 3′ terminus of the pelB signal peptide sequence located in the pET20 vector. The pelB signal peptide can direct FL into the periplasm of *E. coli*, where the signal peptide was cleaved. The recombinant pET20-FL vector was transformed into BL21 (DE3) *E. coli*. A single clone of pET20-FL/BL21 (DE3) was inoculated in 5 mL of ZYM-505 medium with ampicillin (0.1 mg/mL) and cultured for 8 h at 200 rpm at 37 °C [26]. The bacterial culture was then inoculated into 1 L of autoinduction ZYM-5052 medium [26] and induced for 24 h at 200 rpm at 28 °C. Bacteria were collected by centrifugation. The periplasmic fraction in the bacteria containing rhFL was obtained by using the osmotic shock method [27]. The obtained supernatant was mixed at a 1:1 (v/v) ratio with buffer M (40 mM sodium phosphate buffer (PB), 0.6 M NaCl, 60 mM imidazole, pH 8.0). Protein samples were then loaded into an 80 mL Ni–NTA Sepharose column pre-equilibrated with buffer N (20 mM PB, 0.5 M NaCl, 35 mM imidazole, pH 8.0). After sample loading, the column was washed with 5 column volumes (CV) of buffer N and 7 CV of buffer H (20 mM PB, 0.5 M NaCl, 75 mM imidazole, pH 8.0). The target fraction was eluted with buffer T (20 mM PB, 0.5 M NaCl, 800 mM imidazole, pH 6.5). This fraction was mixed at a 1:1 ratio (v/v) with buffer P (40 mM PB, pH 8.0, 1 M ammonium sulfate) and gently stirred for 10 min. This solution was loaded into a 30 mL phenyl Sepharose column pre-equilibrated with buffer Q (20 mM PB, pH 8.0, 0.5 M ammonium sulfate). After sample loading, the column was sequentially equilibrated with 5 CV of buffer Q and washed with 3 CV of buffer R (20 mM PB, pH 8.0, 0.41 M ammonium sulfate), and the target protein was eluted with buffer S (20 mM PB, pH 8.0, 0.13 M ammonium sulfate). This eluted sample was exchanged to buffer T (0.05 M PB, 0.15 M NaCl, pH 7.20) by using an ultracentrifugation tube (10 kDa cutoff value). This buffer exchange step was used to thoroughly remove NH_4_^+^ ions by multiple rounds of ultrafiltration to prepare samples for the conjugation step. Samples from the expression and purification steps were analyzed by polyacrylamide gel electrophoresis (SDS-PAGE) and immunoblotting.

For-FL_ecto_: gttagatatcACCCAGGACTGCTCCTTCCAAC.

Rev-FL_ecto_: cattctcgagTTAGTGGTGGTGGTGGTGGTGGGGCTGCGGGGCTGTCGGGGCTGT.

### Protein conjugation with DM1

Purified rhFL and DM1 were conjugated via the chemical linker SPDP (cleavable) or SMCC (uncleavable). There were two reactive groups located at each end of the linker; for SPDP, they were activated NHS ester and a 2-pyridyldithiol group; for SMCC, they were activated NHS ester and maleimide. At pH 6.5–7.5, the activated NHS ester reacted with the primary amines in the rhFL protein, and 2-pyridyldithiol or maleimide reacted with free sulfhydryl in DM1. The disulfide bond formed between the 2-pyridyldithiol of SPDP and the DM1 drug can be reduced by a disulfide-reducing reagent to release free DM1, while the bond formed between the maleimide of SMCC and DM1 cannot be reduced. For conjugation of DM1 and rhFL via the SPDP linker, the protein concentration of rhFL in buffer T was adjusted to 1 mg/mL. The molar ratio between the chemical linker SPDP and rhFL is 20:1. SPDP stock solution (20 mM in DMA) was added to the rhFL protein solution. The mixture was gently agitated and incubated at room temperature for 1 h. This mixture was then exchanged to buffer TE (buffer T plus 10 mM EDTA) by multiple rounds of centrifugation by using an ultrafiltration tube (cutoff value 3 kDa) to reduce the concentration of SPDP below 1 nM. Before the addition of DM1 stock solution (10 mM in DMA) into FL-SPDP, a 10% (v/v) final concentration of DMA was added into FL-SPDP solution to facilitate DM1 solvation. The DM1 stock solution was then added to the FL-SPDP sample at a molar ratio of 20:1 between DM1 and FL-SPDP. The mixture of FL-SPDP and DM1 reacted for 1 h at room temperature and overnight at 4 °C. After the reaction, the mixture was thoroughly exchanged into buffer T by multiple rounds of centrifugation in an ultrafiltration tube (cutoff value 3 kDa) to reduce the amount of unreacted DM1 below a picomolar concentration of 20. The final product FL-DM1 (denoted FL-SPDP-DM1 thereafter) was stored at − 20 °C. FL-DM1 was analyzed by SDS-PAGE.

For conjugation of DM1 and rhFL via a sulfo-SMCC linker, sulfo-SMCC powder was dissolved in ultrapure water to make up the stock solution (5 mM) immediately before use. Other procedures were the same as those used for the conjugation of SPDP and DM1. The final product, FL-SMCC-DM1, was stored at − 20 °C. FL-SMCC-DM1 was analyzed by reducing SDS-PAGE to characterize the amount of conjugated DM1.

Further, FL-SPDP-DM1 conjugation product was analyzed by LC–MS in Biotech-Pack Scientific Inc. Briefly, FL-DM1 product was separated in ACQUITY UPLC PROTEIN BEH C4 Column by Ultimate 3000 UPLC (Thermo Fisher Scientific, USA), and detected by Q Exactive™ Hybrid Quadrupole-Orbitrap™ Mass Spectrometer (Thermo Fisher Scientific, USA) (see Additional file 1).

### Immunoblotting

Samples were transferred to 0.45 μM PVDF membranes (Millipore). The membrane was washed once with PBST (phosphate-buffered saline, 0.05% v/v Tween-20). Then, the membrane was incubated with blocking buffer (5% w/v BSA in PBST) for 1 h at room temperature. After three washes with PBST, the membrane was incubated with primary antibody (1:1000 dilution) overnight at 4 °C. After three washes, the membrane was incubated with secondary antibody (1:1000 dilution) at room temperature for 1 h. After washing three times with PBST, the membrane was immersed in Pro-light HRP chemiluminescence detection reagent (Thermo Scientific, USA), and the fluorescence was captured using a ChemiDoc system (Bio-Rad, USA).

### Cell signaling assay

Cells were washed with plain culture medium without serum, and 2 × 10^6^ cells were plated in each well in a 12-well plate. After overnight incubation, 5 nM rhFL or 5 nM FL-DM1 (here and elsewhere, the concentration refers to FL-DM1) in plain culture medium was added to the wells. Cells were collected at different time points (5 min, 10 min, 30 min). The cell pellet was resuspended in RIPA solution containing protease and phosphatase inhibitor cocktail and sonicated briefly. The proteins in the cell lysates were separated by SDS-PAGE and analyzed by immunoblotting with anti-p-FLT3, FLT3, p-AKT, AKT, and β-actin (CST, USA) primary antibodies.

### FLT3 internalization assay

Cells (1 × 10^6^) were plated in each well of a 24-well plate and cultured in complete culture medium (plain culture medium plus 10% FBS). Either 100 nM DM1, 5 nM rhFL or 5 nM FL-DM1 was added to the culture medium and incubated for different hours (1 h, 2 h, 4 h) in an incubator. Cells were collected and washed twice with cold washing buffer [1% BSA, 0.03% Proclin-300 in phosphate buffer saline (PBS)] and stained with APC-conjugated anti-FLT3 antibody on ice for 30 min. After three washes with cold washing buffer, the cells were analyzed by a CytoFlex flow cytometer (Beckman, USA). Flow data was analyzed by FlowJo VX software (USA).

### Cell viability, cell cycle and cell apoptosis assay

For the cell viability assay, 3 × 10^4^ cells were plated in each well in a 96-well plate with complete culture medium. For the test groups, different concentrations (from 0.02 nM to 200 nM) of drugs were added to the corresponding wells. The plate was incubated for 3 days before detection. Viable cells were calculated using trypan blue staining with a cytometer. Cell viability was measured by the cell counting kit-8 method.

For apoptosis analysis by western blotting, 3 × 10^6^ cells were cultured with FL-DM1 (15 nM or 30 nM) for 3 days, and the total cells were collected, resuspended in RIPA solution containing protease and phosphatase inhibitor cocktail, and sonicated briefly. Cell lysates were separated by SDS-PAGE and analyzed by immunoblotting. Antibodies recognizing cleaved caspase-3, PARP (full length and cleaved PARP) (CST, USA) or β-actin were used as primary antibodies to detect individual proteins.

For apoptosis analysis by FACS, 1 × 10^6^ cells were treated with FL-DM1 (4 nM or 20 nM) or DM1 (8 nM or 40 nM) for 3 days and then stained with APC-annexin V and propidium iodide (PI) before detection by FACS.

For cell cycle analysis, 2 × 10^6^ cells were treated with FL-DM1 (3 nM, 5 nM, 30 nM) for 48 h. The collected cells were fixed with 70% ethanol overnight at − 20 °C and then stained with PI solution (0.1% Triton X-100, 1 mg/mL DNase-free RNase, 2 µg/mL PI, PBS) before detection by FACS. Flow cytometric analysis was performed by a Cytoflex Flow cytometer (Beckman, USA). Flow data was analyzed by FlowJo VX software (USA).

For competition assay, THP-1 (3 × 10^4^/well) cells were plated in 96-well plate. 10 µg/mL (final concentration) of anti-FLT3 IgG polyclonal antibody (Sino Biological Inc, China) or isotype IgG control was added into wells, and pre-incubated for 2 h. Then FL-DM1 (20 nM) was added into the related wells. Cells were incubated for 48 h before measured by cell counting kit-8 method.

### Statistics

Numerical data are shown as the mean ± standard deviation. Numerical data in each group passed the normality test. Differences between two groups were determined using unpaired two-sample Student’s t-test. Comparison between multiple groups were determined using one-way ANOVA. Significance was analyzed by GraphPad Prism 6.0 (GraphPad Software, Inc.). p < 0.05 indicated the difference was statistically significant.

## Results

### Expression and purification of rhFL protein

**Fig. 1:**
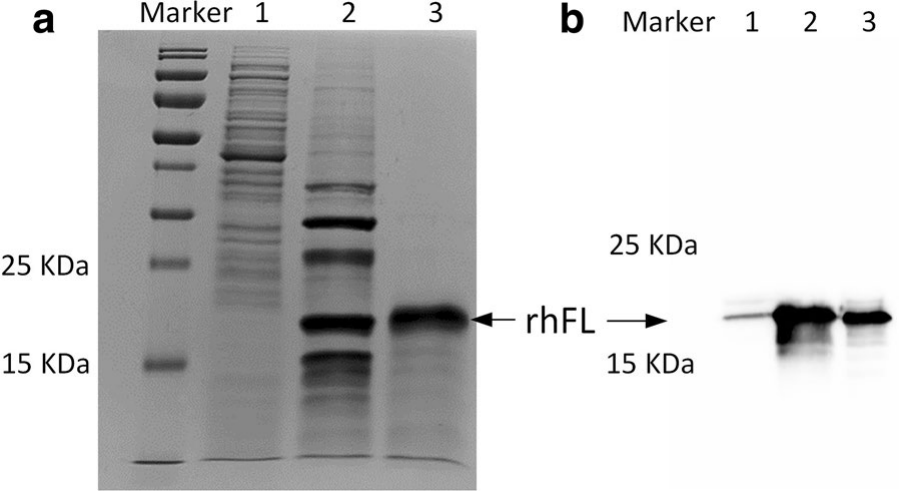
Analysis of the expression and purification of rhFL. **a** SDS-PAGE analysis of the expression and purification of the rhFL protein. Lane 1: periplasmic fraction of induced BL21 (DE3) harboring the pET20-FL plasmid; Lane 2: Ni–NTA Sepharose column elution fraction; Lane 3: phenyl Sepharose column elution fraction. **b** Western blot analysis of rhFL protein. An anti-FL polyclonal antibody was used as the primary antibody. Lane 1: periplasmic fraction of induced BL21 (DE3) harboring the pET20-FL plasmid; lane 2: purified rhFL protein; lane 3: commercial recombinant FL as the positive control

**Fig. 2:**
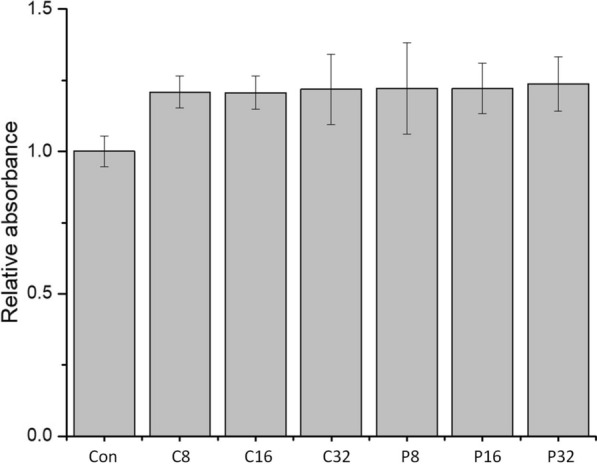
Cell proliferation of THP-1 stimulated by rhFL. THP-1 cells (3 × 10^4^) were plated in culture medium with 1% FBS. rhFL was added to each well at various concentrations. Cells were cultured for 3 days before detection by cell counting kit-8. C8, C16, and C32: cells treated with 8, 16, and 32 ng/mL commercial rhFL, respectively. P8, P16, and P32: cells treated with 8, 16, and 32 ng/mL purified rhFL protein, respectively

The expression of rhFL was induced in ZYM-5052 medium at 28 °C and 200 rpm for 24 h. In our experiments, higher temperatures (30 °C or 37 °C) resulted in reduced target protein expression. The induced bacterial pellets were collected after centrifugation and lysed by the osmotic shock method to release the periplasmic fraction containing rhFL protein. rhFL was purified by two-step purification of Ni–NTA Sepharose and phenyl Sepharose (Fig. 1a). Finally, approximately 0.3 mg purified rhFL with > 90% purity was collected from 1 L of culture medium. The positive rhFL band was detected by immunoblotting using an anti-FL antibody (Fig. 1b). The purified rhFL was thoroughly dialyzed into PBS buffer to remove NH_4_^+^ ions. Our purified rhFL showed bioactivity similar to that of commercial rhFL in stimulating the growth of THP-1 cells (Fig. 2).

### Preparation of the FL-DM1 conjugate

**Fig. 3:**
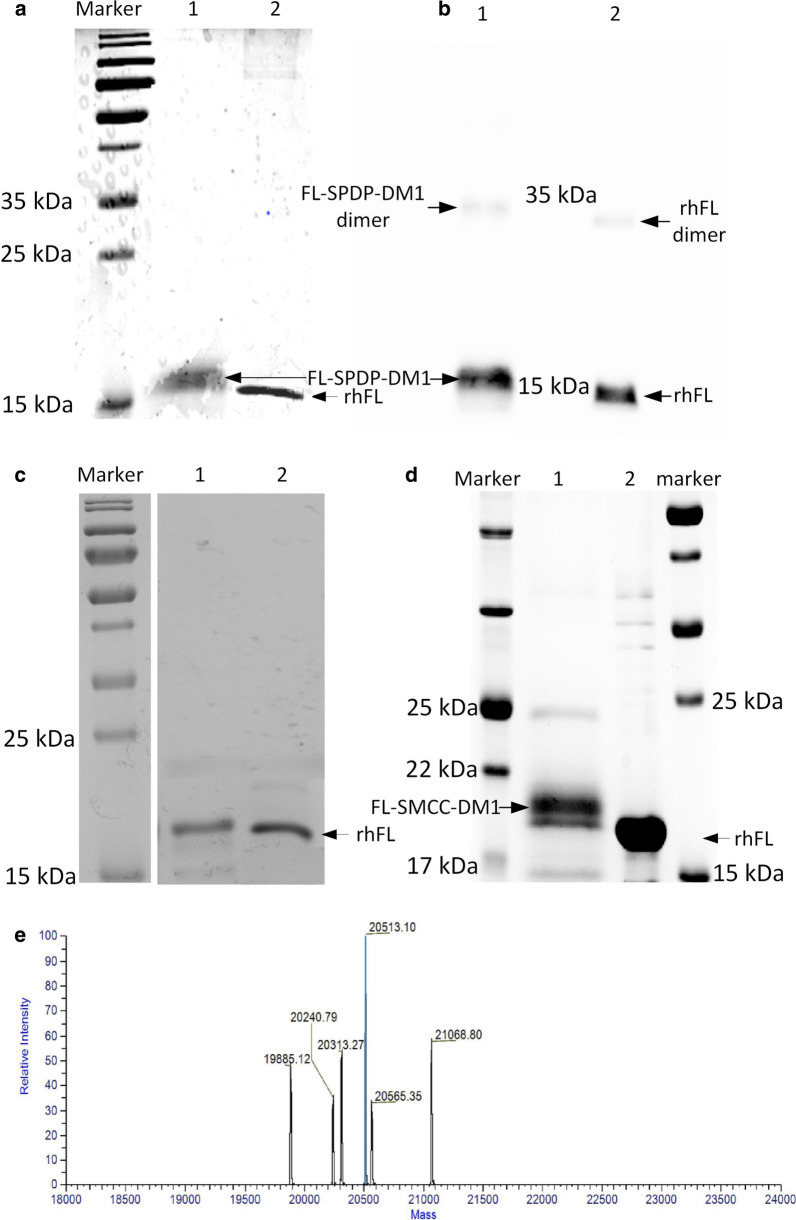
SDS-PAGE analysis of the FL and DM1 conjugation products. **a** Nonreducing SDS-PAGE result of FL-DM1. Lane 1: FL-DM1; Lane 2: rhFL. **b** Western blot analysis of FL-DM1 under nonreducing SDS-PAGE. Lane 1: FL-DM1; Lane 2: rhFL. Primary antibody: anti-His tag antibody. **c** Reducing SDS-PAGE result of FL-DM1. Lane 1: FL-DM1; Lane 2: rhFL. **d** Reducing SDS-PAGE result of FL-SMCC-DM1. Lane 1: FL-SMCC-DM1; Lane 2: rhFL. E. Molecule weight result of FL-DM1 conjugation product analyzed by LC–MS

Purified rhFL was conjugated to DM1 via the chemical linker SPDP. The primary amine in the rhFL protein reacted with the activated NHS ester end of SPDP, and the free sulfhydryl in DM1 reacted with the pyridyl disulfide group of SPDP to form a cleavable disulfide bond. In nonreducing SDS-PAGE, rhFL showed a single band, and the reaction product FL-DM1 showed a smeared band (Fig. 3a), indicating that the FL-DM1 protein comprised a heterogeneous mixture. This phenomenon confirms reports that nonspecific conjugation leads to conjugation products with different amounts of conjugated DM1 drug [28, 29]. Western blot result showed FL-DM1 and FL-DM1 dimer both had higher molecule weight band than FL and FL dimer (Fig. 3b). Under reducing conditions, the disulfide group between SPDP and DM1 in FL-DM1 was reduced to a free sulfhydryl group. Therefore, DM1 was released from FL-DM1, leaving rhFL-SPDP intact. Thus, the FL-DM1 sample showed a slightly higher band than the rhFL sample in reducing SDS-PAGE (Fig. 3c). These results demonstrated that DM1 was successfully conjugated to rhFL. The protein recovery rate was approximately 40%.

Nonreducing SDS-PAGE is not suitable to calculate protein molecule weights. To primarily characterize the molecular weight of FL-DM1, an uncleavable SMCC linker, which reacts with the same moieties of FL and DM1 as the SPDP linker, was used to generate the FL-SMCC-DM1 drug conjugate. Based on the result of reducing SDS-PAGE (Fig. 3c), FL had a theoretical molecular weight of 19.1 kDa (with 3 extra amino acids located at its N-terminus and 6 histidines at its C-terminus) and produced a single band, while FL-SMCC-DM1 produced a smeared band, representing a heterogeneous mixture. FL-SMCC-DM1 migrated at approximately 21 kDa (Fig. 3d), and one conjugated molecule of SMCC-DM1 was approximately 960 Da. Therefore, an average of 2 molecules of SMCC-DM1 were conjugated to one molecule of rhFL by using this conjugation method. As based on our results and published articles [30, 31], nonspecific conjugation produces heterogeneous products, one protein molecule is conjugated with a few molecules of cytotoxic drugs.

LC–MS analysis showed the general molecule weight distribution of FL-SPDP-DM1 reaction product (Fig. 3e). Result confirmed that reaction product was mixture. Based on analysis, 1–3 molecule(s) of SPDP-DM1 was conjugated to one molecule of rhFL, and the main proportion of the reaction product was one molecule of rhFL conjugated with 2 molecules of SPDP-DM1.

### FL-DM1 can be efficiently internalized by FLT3-expressing cells

**Fig. 4:**
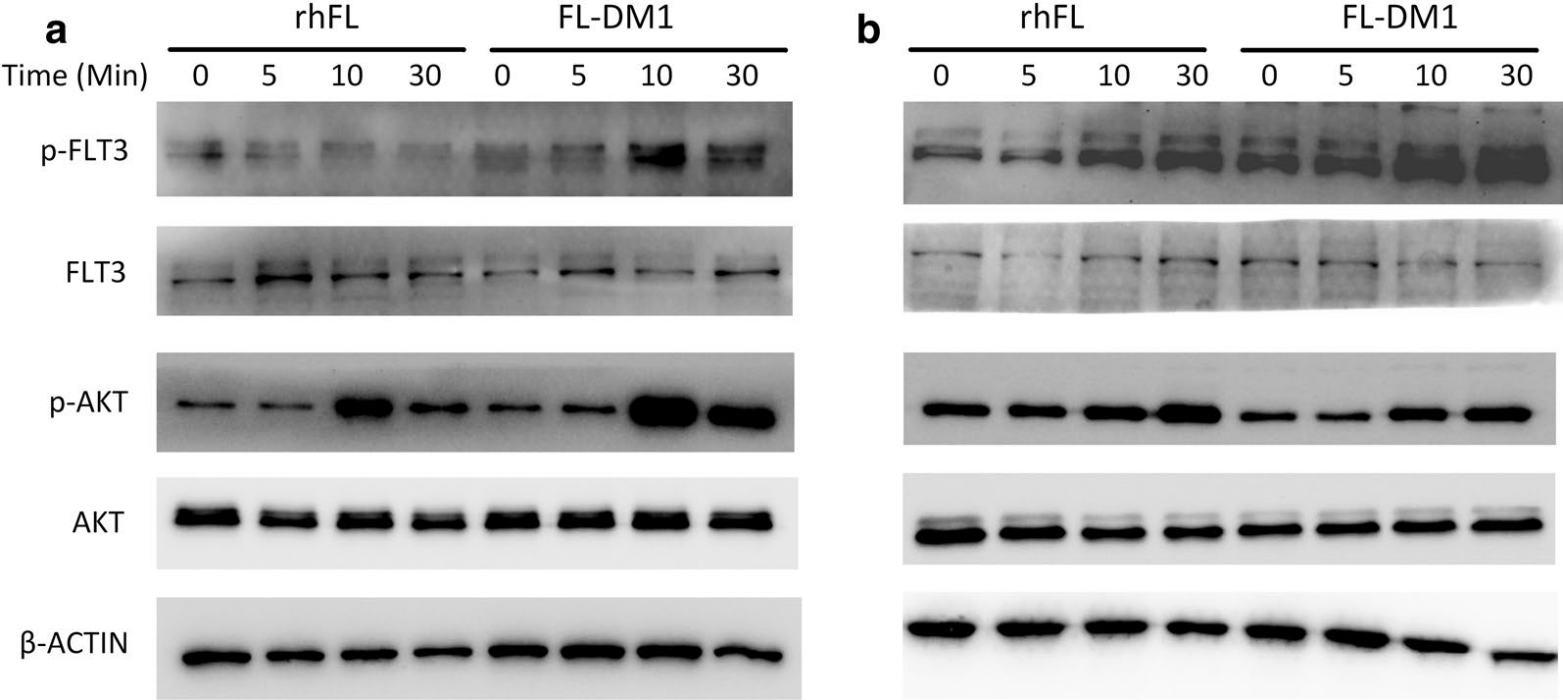
Immunoblot analysis of the phosphorylation levels of FLT3 and AKT in MV-4-11 cells (**a**) and THP-1 cells (**b**) treated with rhFL (5 nM) versus FL-DM1 (5 nM). The total levels of FLT3 and AKT were used as controls

DM1 targets proliferating cells by depolymerizing microtubules through binding at the vinca binding site of tubulin [32]. However, DM1 modifies the conformational structure of conjugated rhFL, which could impair the bioactivity of rhFL and thus may hinder the FLT3 internalization induction ability of rhFL. To assess this effect, the phosphorylation of FLT3 and the downstream signaling protein AKT were measured in AML cells treated with rhFL or FL-DM1. Our data showed that treatment with rhFL or FL-DM1 significantly increased the phosphorylation of FLT3 and AKT in THP-1 and MV-4-11 cells (Fig. 4). These results demonstrated that FL-DM1 was as functional as rhFL in activating the FLT3 signaling pathway in FLT3-expressing leukemia cells, indicating that FL-DM1 retained the bioactivity of FL.

**Fig. 5:**
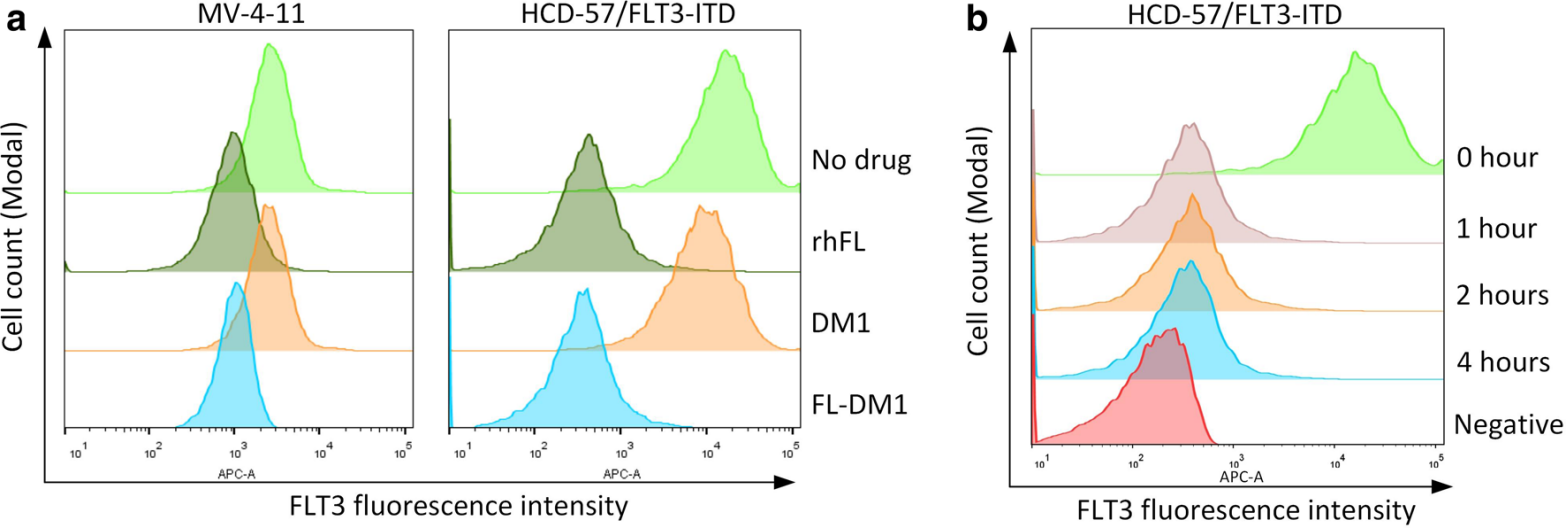
FACS analysis of the surface FLT3 expression level. **a** Cells treated with rhFL, DM1 or FL-DM1 for 1 h. DM1: 100 nM. Recombinant hFL: 5 nM. FL-DM1: 5 nM. **b** Cells were treated for different time (1 h, 2 h, and 4 h) with FL-DM1 (5 nM)

FL can be internalized into cells when binding to FLT3, providing a mechanism by which FL-DM1 specifically targets FLT3-expressing cells. To investigate the internalization of FL-DM1, the surface expression of FLT3 was detected in leukemia cells treated with drugs. MV-4-11 and HCD-57/FLT3-ITD cells were treated with FL, FL-DM1, or DM1 for different times. The surface expression of FLT3 in both cell lines decreased to the similar extent in the FL- and FL-DM1-treated groups but remained unchanged in the DM1 treatment group (Fig. 5a). We also tested the FLT3 internalization rate under FL-DM1 treatment. The membrane FLT3 level changed little from 1 to 4 h after addition of FL-DM1 (Fig. 5b). These results indicated that FL-DM1 had uncompromised internalization induction ability, and can be quickly and efficiently absorbed into cells.

### FL-DM1 inhibits FLT3-positive acute leukemia cells

**Fig. 6:**
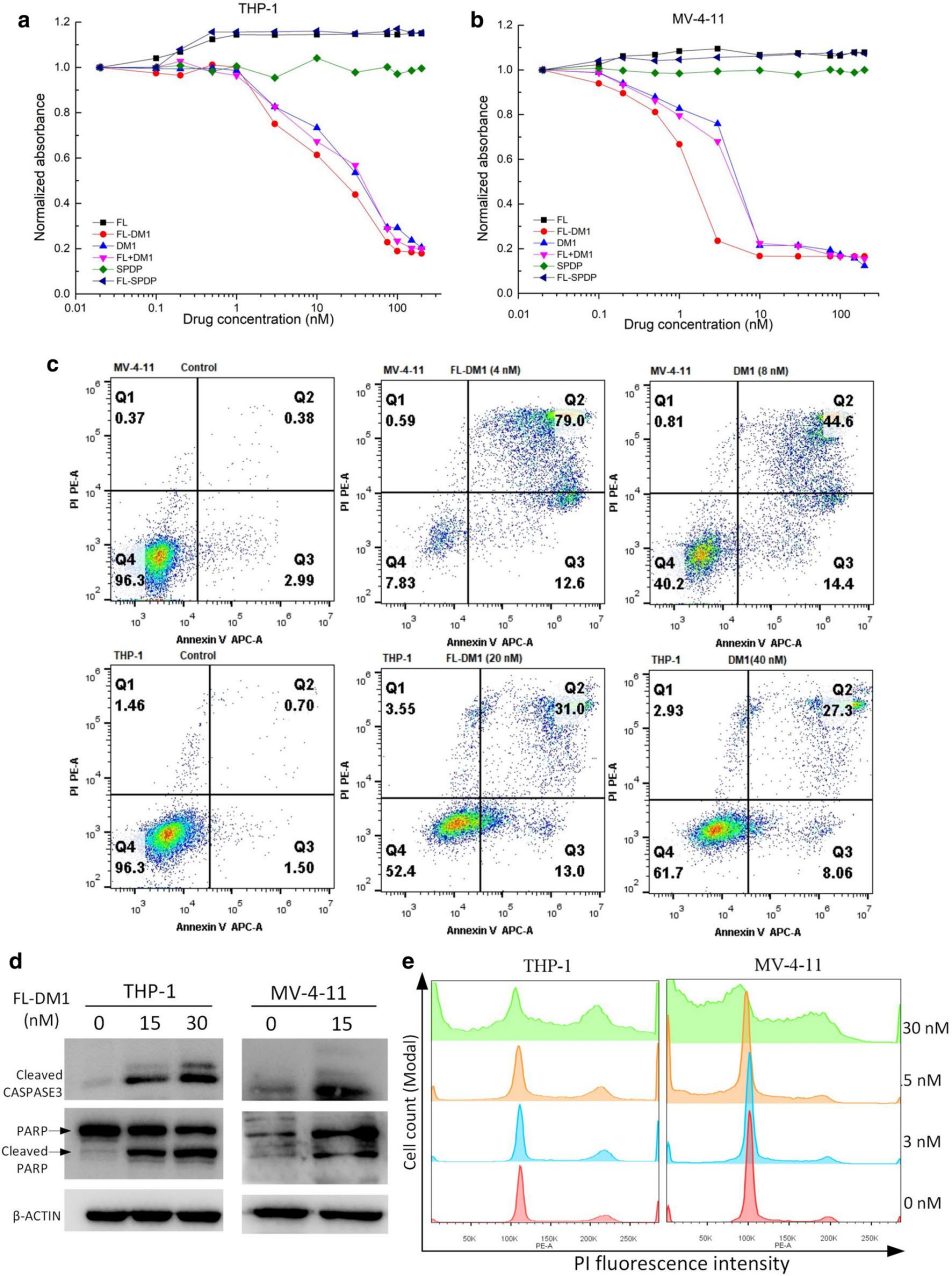
Cell viability, apoptosis and cell cycle arrest assays of MV-4-11 and THP-1 cells treated with FL-DM1. **a** Viability assay of THP-1 cells treated with various concentrations of drugs. FL + DM1 group: unconjugated FL and DM1 with the same concentration. **b** Viability assay of MV-4-11 cells treated with various concentrations of drugs. FL + DM1 group: unconjugated FL and DM1 with the same concentration. **c** FACS analysis of apoptosis of MV-4-11 and THP-1 cells under FL-DM1 or DM1 treatment. **d** Western blotting analysis of activated caspase-3 and PARP in MV-4-11 and THP-1 cells under FL-DM1 treatment. E. Cell cycle analysis of MV-4-11 and THP-1 cells treated with FL-DM1

The cytotoxicity of FL-DM1 was assessed in the FLT3-positive AML cell lines MV-4-11 and THP-1. In the cell viability assay, FL-DM1 showed an inhibitory effect on THP-1 and MV-4–11 cells in the nano-molar concentration range (Fig. 6a, b). The IC_50_ of FL-DM1 for THP-1 and MV-4–11 cells were 12.9 nM and 1.1 nM, which were calculated by dose–response curve using GraphPad. FACS analysis revealed that 90% of MV-4–11 cells were induced apoptosis in the FL-DM1-treated group (4 nM) compared with that in the control group. In THP-1 cells, approximately 44% cells were induced apoptosis in the group treated with FL-DM1 (20 nM) compared with that in the control group (Fig. 6c). Of note, DM1 as a single agent had similar effect on these two cells. Western blotting analysis of AML cells treated with FL-DM1 showed increased expression of cleaved caspase-3 and cleaved PARP, indicating obvious cell apoptosis (Fig. 6d). In addition, DM1 arrested the cell cycle at G2/M phase. FL-DM1 also showed the same inhibitory mechanism in THP-1 and MV-4–11 cells (Fig. 6e).

### FL-DM1 selectively targets FLT3-expressing leukemia cells

**Fig. 7:**
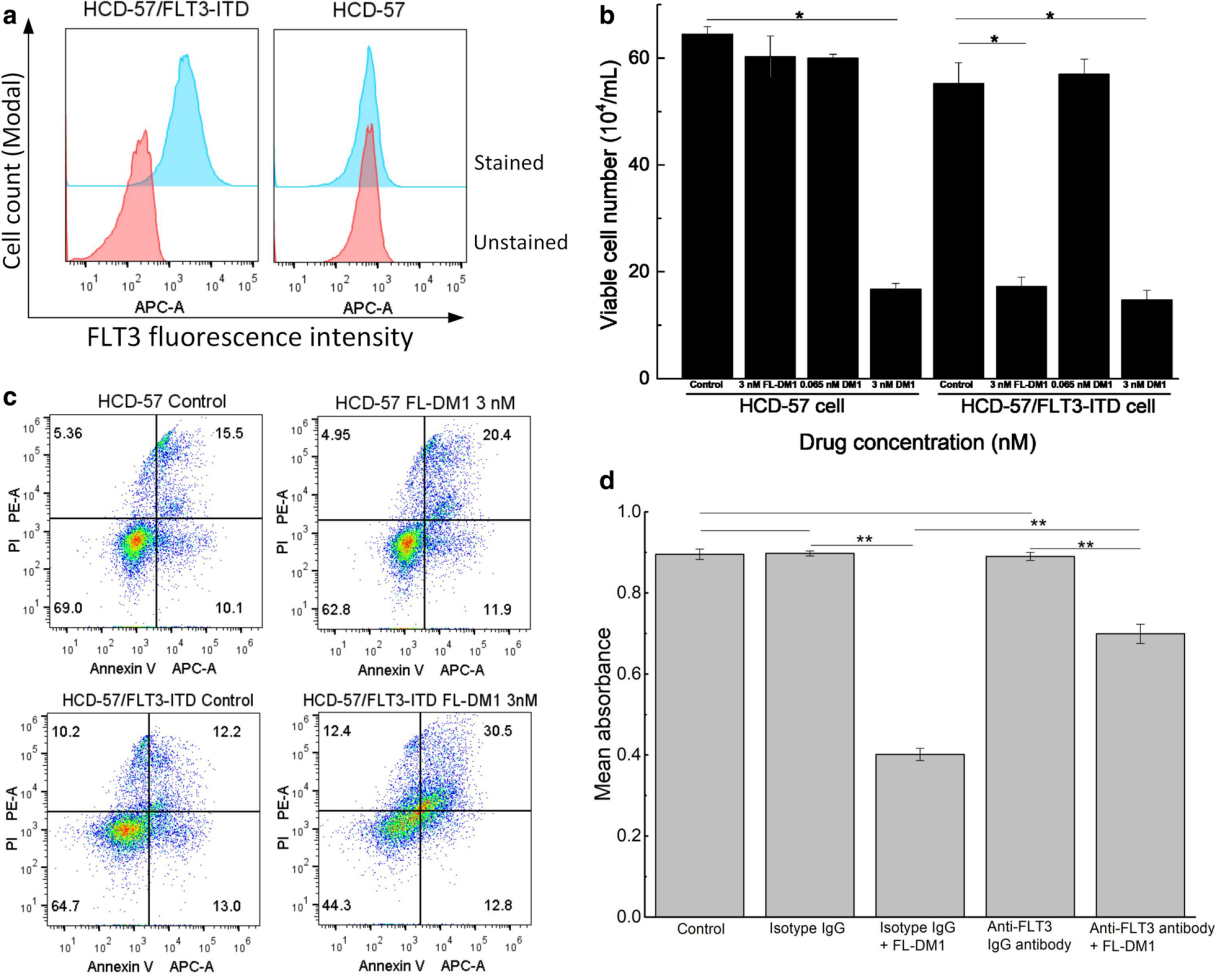
FL-DM1 selectively targets HCD-57 cells expressing FLT3. **a** Surface FLT3 expression levels in HCD-57 and HCD-57/FLT3-ITD cells. **b** The viability of HCD-57 and HCD-57/FLT3-ITD cells treated with 3 nM FL-DM1 or 3 nM DM1, *: p < 0.05. **c** Apoptosis of HCD-57 and HCD-57/FLT3-ITD cells treated with 3 nM FL-DM1. **d** FL-DM1 cytotoxicity on THP-1 cells under co-incubation with anti-FLT3 polyclonal antibody. FL-DM1: 20 nM, anti-FLT3 polyclonal antibody or isotype IgG: 10 µg/mL. **: p < 0.01

We used FLT3-negative HCD-57 cells to evaluate the selectivity of FL-DM1. HCD-57 cells are murine erythroleukemia cells and require exogeneous erythropoietin (EPO) for survival. When transformed by a retrovirus vector carrying the human FLT3-ITD gene, HCD-57/FLT3-ITD acquired the ability to proliferate without EPO (Fig. 7a). A cell viability assay was used to determine the inhibitory potency of FL-DM1 and DM1. FL-DM1 (3 nM) potently inhibited the viability of HCD-57/FLT3-ITD cells, but it had essentially no effect on the parental HCD-57 cells. DM1 at 3 nM strongly inhibited the growth of both HCD-57/FLT3-ITD and parental HCD-57 cells (Fig. 7b). The data indicated that FL-DM1 selectively targeted FLT3-expressing HCD-57/FLT3-ITD cells and inhibited their proliferation, while free DM1 inhibited both cells irrespective of the FLT3 expression status. We further used FACS to measure apoptosis induction by FL-DM1 in HCD-57/FLT3-ITD and parental HCD-57 cells. When treated with 3 nM FL-DM1, HCD-57 cells showed no significant increase in apoptosis compared with the HCD-57 control group, while the HCD-57/FLT3-ITD group showed a more than one-fold increase in late apoptotic cells compared with the control group (Fig. 7c). These results demonstrated that FL-DM1 selectively inhibited FLT3-expressing cells, while free DM1 had no selectivity.

Internalization of FL-DM1 via FLT3 receptor was also validated by competition assay. Either anti-FLT3 antibody or isotype IgG control, had little effect on proliferation of THP-1 cells compared with control. FL-DM1 (20 nM) had cytotoxicity towards THP-1 cells. But when co-incubated with anti-FLT3 polyclonal antibody, FL-DM1 (20 nM) showed decreased cytotoxicity towards THP-1 cells (Fig. 7d). The antibody may hinder the binding of FL-DM1 to FLT3, and thus reduced the quantity of FL-DM1 that entered into cell cytosol. Result validated that FL-DM1 needed the assistance of FLT3 receptor to enter the cytosol of THP-1 cells.

### FL-DM1 induces apoptosis in primary FLT3-positive leukemia cells ex vivo

**Fig. 8:**
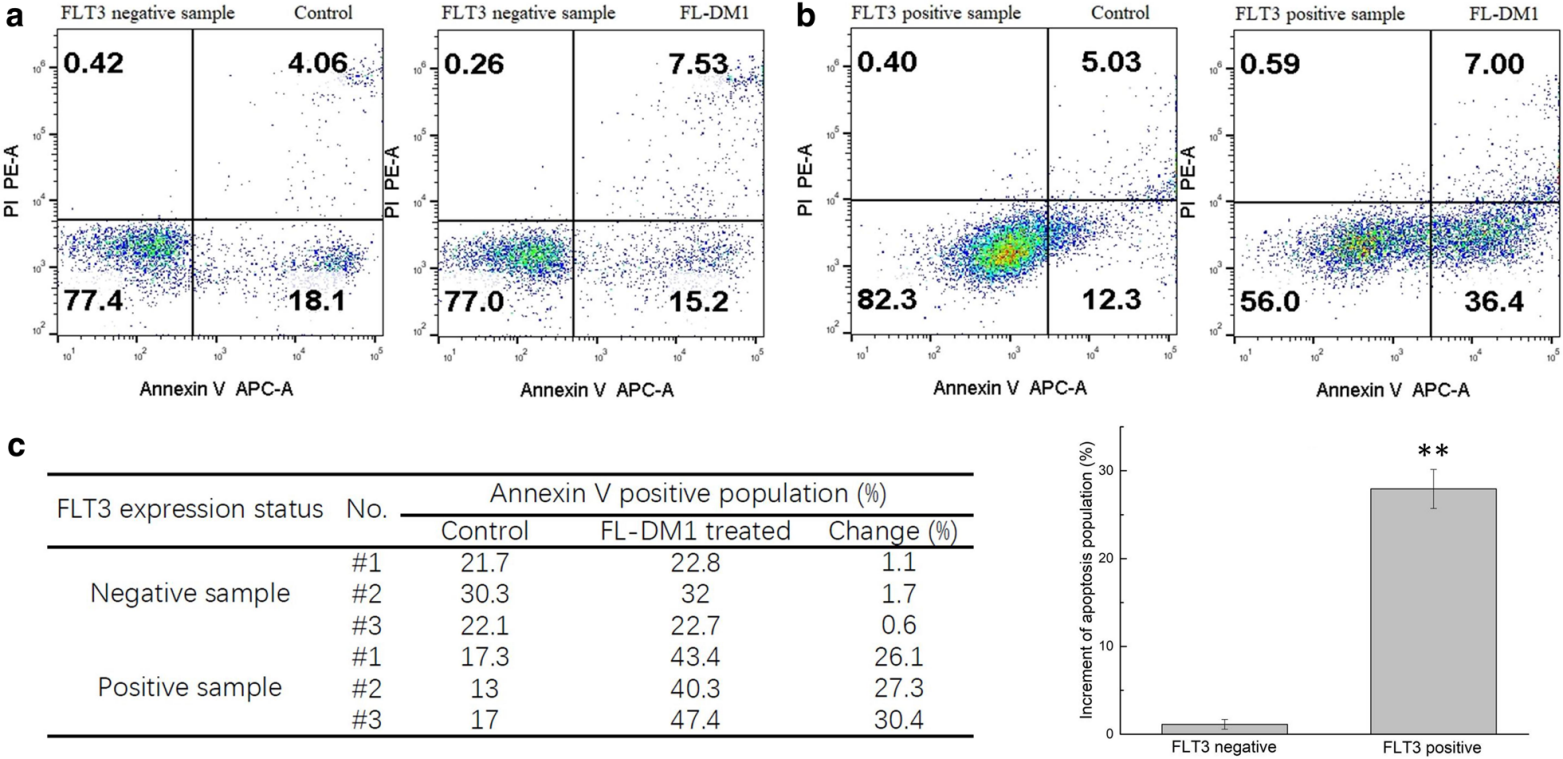
Analysis of FL-DM1 on primary AML cells. **a** Typical FLT3-negative primary AML cells treated with FL-DM1 (10 nM). **b** Typical FLT3-positive primary AML cells treated with FL-DM1 (10 nM). **c** Statical analysis of Annexin V positive population increment in two groups (FLT3-negative group and FLT3-positive group) after FL-DM1 (10 nM) treatment, **: p < 0.01

Primary AML cells from patients were treated with FL-DM1 to further evaluate the cytotoxicity of FL-DM1. Cells (2 × 10^6^) were cultured in complete tissue culture medium (IMDM plus 10% FBS) with FL-DM1 (10 nM) for 40 h. The cell apoptosis rate was measured by FACS. In the FLT3-negative samples, FL-DM1 had no cytotoxic effect compared with the control group (Fig. 8). In FLT3-positive samples, FL-DM1 induced at least two-fold increase in apoptosis compared with the control (Fig. 8). These results further demonstrated that FL-DM1 had selectivity and cytotoxicity towards primary AML cells with FLT3 expression ex vivo.

## Discussion

The FL/FLT3 signaling pathway functions as an important prosurvival signaling pathway in the development and progression of AML. Gain-of-function FLT3 mutations were found in 30–40% of AML patients [5], and FLT3-ITD is an independent marker for poor prognosis [33]. In AML patients treated with chemotherapy, the FL concentration can increase to 3.25 ng/mL and impede the efficacy of FLT3 kinase inhibitors [34]. FL/FLT3 pathway-directed STAT5 activation leads to increased expression of MCL-1, a hallmark biomarker of leukemia stem cells [35]. Therefore, targeting FL/FLT3 is one of most attractive therapeutic strategies in patients with AML. In this article, our FL drug (DM1) conjugate showed promise as a new drug for the treatment of FLT3-positive AML patients.

In our study, recombinant human FL was produced as a nonglycosylated protein in the *E. coli* expression system. It is expressed as a bioactive protein in the periplasm of *E. coli* with the assistance of chaperones [36]. The purified rhFL was functional, as shown in the proliferation assay and FLT3 internalization assay. This method is feasible for the production of rhFL, and high-density fermentation can be used to obtain target proteins in the periplasm of *E. coli* [37, 38]. Compared with the anti-FLT3 inhibitory antibody, FL has a high affinity (200–500 pM) for the FLT3 receptor, which is comparable to the affinity of the IMC-EB10 inhibitory antibody [39]. Anti-FLT3 antibodies need extensive development by using hybridoma technology or screening with a human Fab phage display library [40]. rhFL can be cost-effectively and conveniently produced from recombinant *E. coli* and is capable of delivering cytotoxic drugs into FLT3-expressing primary AML cells and AML cell lines, as we demonstrated in this work.

Cytotoxicity of the free drug DM1 relies on its transport across the membrane by membrane transporters/pumps, while the cytotoxicity of FL-DM1 relies on the membrane FLT3 expression level, the degradation of FL-DM1, the release of active DM1 in the cytosol and the nature of targeted cells [29, 41, 42]. The group of Dr. Lewis reported that a trastuzumab-DM1 conjugate targeting the HER2 receptor was more efficient than nonspecific uptake of free DM1 drug in cells highly expressing the HER2 receptor (SK-BR-3 and BT-474 cells); however, in cells with normal or absent expression of the receptor (MCF-7 and MDA-MB-468 cells), trastuzumab-DM1 was less efficient than the free DM1 [41]. In our cell-based assay, IC_50_ value of FL-DM1 (2.2 nM conjugated DM1) was lower than free DM1 (4.0 nM) in MV-4-11 cells. IC_50_ value of FL-DM1 (25.8 nM conjugated DM1) was comparable with that of free DM1 (26.0 nM) in THP-1 cells. For HCD-57/FLT3-ITD cells, in which 3 nM FL-DM1 (6 nM conjugated DM1) showed approximately equal cytotoxicity compared with 3 nM free DM1. This minor cytotoxicity discrepancies between FL-DM1 and DM1 on cells may result from surface expression of FLT3 and the nature of targeted cell.

The advantage of FL-DM1 lies in its selectivity and effective delivery of DM1 into FLT3-expressing AML cells. Although free DM1 is highly cytotoxic towards proliferating cells (IC_50_ value between 10^–11^ and 10^–9^ M), it can cause severe side effects due to the nonselectivity, which limits the clinical application of DM1 in the treatment of cancer [43]. It is unsuitable for direct use in the treatment of cancer. Protein-conjugated drugs have the advantages of the cytotoxicity of DM1 and the selectivity of receptor-targeting proteins [44] and can evade drug resistance conferred by multidrug transporters [45]. In our case, the IC_50_ of FL-DM1 was lower than free DM1 in AML cell lines, and it was below 13 nM, which made further drug testing possible.

Similar to anti-FLT3 therapeutic antibodies, FL-DM1 targeted both mutant FLT3- and wild-type FLT3-expressing AML cells, as demonstrated above. Antibodies involved in ADC always inhibit the signaling pathway mediated by their targeted receptor, while the wild-type FL in FL-DM1 temporally stimulates FLT3 phosphorylation and downstream AKT. For protein-conjugated drugs, the killing effect of protein-conjugated drugs tends to be rapid and is attributed to chemical drugs. Whether drug vehicles are inhibitory or stimulatory has not been confirmed. Whether the activating function of FL in FL-DM1 is advantageous or disadvantageous still needs to be examined. Otherwise for healthy cells, negative feedback mechanism can efficiently suppress the aberrantly activated FLT3 signaling pathway rendered by exogenous FL. The fact that interleukin-2 in denileukin diftitox (Ontak®) is an agonist ligand of the interleukin-2 receptor in the treatment of cutaneous T-cell lymphoma may support the potential therapeutic use of FL-DM1 [46]. Furthermore, site-specific mutagenesis is a strategy to improve or alter the bioactivity and pharmacokinetic properties of recombinant therapeutic proteins [47, 48]. Several mutations have been reported to impact the activity of rhFL [49]. The H8Y mutein and K84E/Q122R mutein of FL show a three-fold increase in activity compared with wild-type FL, and the H8R mutation in FL shows little activity in cell-based assays [49]. By expressing muteins with decreased or increased stimulatory activities and comparing the cytotoxicity of FL-DM1 muteins and wildtype FL-DM1 in vitro and in vivo, we can choose a suitable form of FL that shows better cytotoxicity and pharmacological properties.

In clinic, targeting FLT3 has been proven to be an efficacious strategy for the treatment of AML [5]. Midostaurin with daunorubicin and cytarabine can significantly prolong overall and event-free survival among AML patients with FLT3 mutations [12]. Specific FLT3 tyrosine kinase inhibitors only target AML cells with mutated FLT3, and FL-DM1 can target AML cells with both mutant and wild-type FLT3 and therefore has broader applications. Free DM1 drug has 10–200-fold higher cytotoxicity than vincristine [50], and conjugated FL-DM1 may show reduced side effects compared to free DM1 and deliver more potent DM1 into target cells [41]. FL-DM1, when used alone or in combination with other drugs, may provide a therapeutic advantage to AML patients with FLT3 expression.

Based on these results, rhFL can be expressed as a soluble protein in *E. coli*, which can be used as a promising specific vehicle to deliver toxic chemical reagents into FLT3-expressing AML cells. Further work will focus on in vivo efficacy experiments and the screening of suitable FL mutein-DM1 conjugates.

## Supplementary Information

**Additional file 1.** LC-MS materials, methods and results: S1. Chemicals and Instrumentation; S2. Sample Preparation; S3: LC-MS Analysis method; S4: LC-MS analysis results

## Data Availability

The datasets used and/or analyzed during the current study are available from the corresponding author upon reasonable request.

## Abbreviations

AML: Acute myeloid leukemia
ADC: Antibody drug conjugate
CV: Column volume
DM1: Emtansine
DMA: Dimethylacetamide
EPO: Erythropoietin
FL: FLT3 ligand
FLT3: FMS-like tyrosine kinase 3
FLT3-ITD: FLT3 internal tandem duplication mutant
HCD-57/FLT3-ITD: HCD-57 cells transformed with FLT3-ITD
HER2: Human epidermal growth factor receptor 2
IC_50_: Half maximal inhibitory concentration
PB: Sodium phosphate buffer
PBS: Phosphate buffer saline
PBST: Phosphate-buffered saline, 0.05% (v/v) Tween-20
PI: Propidium iodide
PTB−: Phosphotyrosine binding domain−
SH2−: Src homology domain−
SMCC: Sulfosuccinimidyl-4-(*N*-maleimidomethyl)cyclohexane-1-carboxylate
SPDP: *N*-Succinimidyl 3-(2-pyridyldithio)propionate
