# Supplementary material for: Expression of a recombinant FLT3 ligand and its emtansine conjugate as a therapeutic candidate against acute myeloid leukemia cells with FLT3 expression
Source: Microb Cell Fact. 2021 Mar 10;20:67. doi: 10.1186/s12934-021-01559-6 (PMC7948335; doi:10.1186/s12934-021-01559-6)
Supplement: Supplementary file 1 — Additional file 1. LC-MS materials, methods and results: S1. Chemicals and Instrumentation; S2. Sample Preparation; S3: LC-MS Analysis method; S4: LC-MS analysis results [file 12934_2021_1559_MOESM1_ESM.docx]

Additional material

**LC-MS materials, methods and results**

1. Chemicals and Instrumentation

Formic acid (FA), acetonitrile (ACN), methanol, were purchased from Sigma (St. Louis, MO, USA). Ultrapure water was prepared from a Millipore purification system (Billerica, MA, USA). An Ultimate 3000 coupled with a Q Exactive™ Hybrid Quadrupole-Orbitrap™ Mass Spectrometer (Thermo Fisher Scientific, USA) with an ESI nanospray source.

2. Sample Preparation

2.1 Take the sample and centrifuge at 12000 rcf at 4°C for 10 min, collect the supernatant.

2.2 Transfer the supernatant to the sample tube and wait for LC-MS analysis.

3. LC-MS Analysis

3.1 UPLC

Ultimate 3000 (Thermo Fisher Scientific, USA)

Column：ACQUITY UPLC Protein BEH C4 Column (300Å, 1.7 μm, 2.1 mm×50 mm);

Mobile phase: A: 0.1% formic acid in water; B: 0.1% formic acid in acetonitrile.

Total flow rate: 0.300 mL/min

LC linear gradient: from 5% to 5% B for 2 min, from 5% to 35% B for 1 min, from 35%

to 95% B for 7 min, from 95% to 95% B for 4 min, from 95% to 5%

B for 0.1 min, eluting with 5% B for 0.9 min.

3.2 Mass spectrometry

Q Exactive™ Hybrid Quadrupole-Orbitrap™ Mass Spectrometer (Thermo Fisher

Scientific, USA)

Mass range: m/z 400-5000

Output Mass: M

S/N Threshold: 3

Rel. Abundance Threshold (%): 0

Charge Range: 2-100

Min. Num Detected Charge: 2

4. Results:

A. Mass spectrometry result of FL-DM1 conjugation product

B. Deconvoluted molecule weight of FL-DM1 conjugation product

Table S1. Data and parameters related to the calculation of FL-DM1 molecule weight.
